# The State of Lunacy in Scotland

**Published:** 1861-07

**Authors:** 


### Art. VIII.—THE STATE OF LUNACY IN SCOTLAND.

The third Annual Report of the Commissioners in Lunacy for Scotland, on the condition and management of Lunatics and Lunatic Asylums in that kingdom, now lies before us. From it we learn that in 1859, as in 1858, there was a "considerable increase" in the amount of pauper lunatics. Thus on the 1st of January, 1858, the number returned amounted to 4737, on the 1st of January, 1859, to 4980, and on the 1st of January, 1860, to 5226, showing an increase of 243 in 1858, and of 246 in 1859.

The following table sets forth the number of known insane in Scotland on the 1st of January, 1860, and the mode in which they were domiciled. It is important to note that the number of lunatics in private houses is only approximately estimated:—

| | | | | Private. | | | Pauper. | | |
|---|---|---|---|---|---|---|---|---|---|
| | Male. | Female. | Total. | Male. | Female. | Total. | Male. | Female. | Total. |
| In Public Asylums... | 1355 | 1277 | 2632 | 402 | 371 | 773 | 953 | 906 | 1859 |
| ,, Private ,, | 349 | 503 | 852 | 84 | 112 | 196 | 265 | 391 | 656 |
| ,, Poorhouses......... | 349 | 517 | 866 | ... | 2 | 2 | 349 | 515 | 864 |
| ,, Private Houses .. | 1869 | 1865 | 3734 | 1041 | 846 | 1887 | 828 | 1019 | 1847 |
| Total............ | 3922 | 4162 | 8084 | 1527 | 1331 | 2858 | 2395 | 2831 | 5226 |

From this table it would appear that of 8084 lunatics in Scotland, 2858 are supported by private funds, and 5226 by parochial rates. From a comparison of these numbers with those of the preceding year, it is ascertained that the increase in the number of the insane is restricted to pauper lunatics. Thus the corresponding numbers on the 1st of January, 1859, were 2898 and 4980. On the other hand the number of private patients had diminished. "This result," the Commissioners remark, "is probably in great measure due to the transfer of a number of the indigent insane from the class of private patients to that of paupers."

The difficulty of determining what constitutes a lunatic in the eye of the law, complained of by the Commissioners in previous Reports, still impedes their efforts in carrying the law into effect. They are authorized, for example, to "permit a parochial board to dispense with the removal of any pauper lunatic to an asylum, and to provide for him in such other manner, and under such regulations, as to inspection or otherwise, as they may sanction." They have, therefore, granted permission to upwards of 2000 cases to remain under private care, dispensing with their removal to asylums. But the statute defines a lunatic to be, "any mad or furious or fatuous person, or person so diseased or affected in mind as to render him unfit, in the opinion of competent medical persons, to be at large, either as regards his own personal safety, or the safety of the persons and property of others, or of the public." This definition renders it doubtful whether insane paupers, exempted from removal to an asylum, are to be reckoned as lunatics. Consequently it is doubtful, also, whether the Board of Supervision for the Relief of the Poor or the Board of Lunacy are responsible for the proper care and treatment of lunatic paupers not in asylums. The Board of Lunacy have hitherto acted on the belief that the responsibility lay with them, but the Commissioners observe, "practical difficulties are constantly occurring, which make it extremely desirable that the statute should explicitly declare that insane paupers, exempted from removal, belong to the category of pauper lunatics. All dubiety on this point would at once be removed, by making the definition of lunacy include any person certified by two competent medical men to be 'a lunatic, an insane person, an idiot, or a person of unsound mind.'"

One of the most effective means at the command of the Commissioners for improving the condition of pauper lunatics, is to require their removal to an asylum if they are not satisfactorily cared for by the parochial authorities. But the Commissioners complain that "their efforts in this direction are occasionally defeated by the decision of the Sheriff, that the patient, though undoubtedly of unsound mind, is not a lunatic in the statutory sense, from the medical certificates containing no statement of facts showing him to be dangerous either to himself or others." Thus it happens from time to time that cases unfit to be at large on other grounds, and who are utterly neglected by those who have the care of them, cannot be properly dealt with on account of the Sheriff's decision. There is, moreover, a total want of uniformity in the practice of different Sheriffs in confirming the order for the admission of a lunatic to an asylum. While, on the one hand, some adhere strictly to the letter of the law, others ratify the medical certificate as a mere form. Evils arise out of both practices, for if by the one lunatics who, if justly treated, should be confined in an asylum, are kept out, by the other persons said to be lunatics are at times admitted on very insufficient evidence. But the Commissioners reiterate an opinion expressed in former Reports, that—

"Less evil is likely to result from the Sheriff's accepting, as proof of insanity, the simple certificates to this effect of two qualified men, than from his refusing to receive them, unless facts plainly proving the existence of insanity are, at the same time, stated. A medical man, we remarked, may, from the manner, appearance, and conduct of a patient, be thoroughly convinced of his insanity, and may nevertheless fail, by any statement of facts, to convey the same conviction to another person; and this difficulty will be greatest in the incipient stages of the malady. No careful observer can have failed to notice that almost all the murders and suicides committed by lunatics take place at the outset of the malady, before the symptoms are sufficiently developed to enable medical men to grant certificates in such form as would satisfy the Sheriff."

Thus, of two cases cited, the Sheriff's order was granted in one on certificates containing scarcely any evidence of insanity, while it was refused in the following instance, in which the facts stated in the certificates were, to say the least, equally strong. In this case it was stated that the patient had "an excited, restless expression, is very talkative, and complains of weariness of life," and that "her manner and conversation indicate the weak mind of old age." She was reported, also, on the statement of others, to be sleepless, affected with delusions, garrulous, querulous, advanced in years, frail in mind and weak in body, and requiring constant care and supervision. The order for admission to an asylum was refused, and the patient was in consequence placed in the ordinary wards of a workhouse. "The result was, that she rose from her bed during the first night, threw open the window, flung herself out, and was killed on the spot."

"Experience," the Commissioners state, "has convinced us that it will be impossible to introduce a uniform procedure as regards orders so long as the Sheriff seeks, in his judicial capacity, to determine whether the medical certificates afford sufficient evidence of the existence of insanity. We would therefore suggest that the Sheriff should, as a matter of course, grant his order for the admission of patients on the simple certificates of two qualified medical men, that the patient is insane and a proper person to be detained in an asylum; and that it should be the duty of the Commissioners to examine the certificates, and to call for their amendment when defective; or to require the discharge of the patient when the evidence of insanity appeared imperfect and could not be substantiated. It must frequently happen that stringent statutory directions as to the disposal of lunatics cannot be adhered to without great risk of accidents, and we are therefore of opinion, that it is not advisable to administer the law of lunacy as stringently, or with as little latitude, as if it formed part of the criminal code. Accordingly, we consider that, in in the following case, the Sheriff acted rightly in granting his order for the reception of the patient into an asylum, although no facts indicating insanity are stated in the certificates, as having been observed by the medical men themselves. All the evidence of its existence rests on the statements of the patient himself. The first medical man merely certifies that the patient 'complains of his head, and says that he must be punished, and that he has frequently thought of destroying himself;' while the second says, that 'in conversation he has told me that he is frequently seized with an impulse to jump into a well and drown himself, and that he has been subject to this feeling since Christmas last, and long before.' Here the whole evidence of insanity, so far as the facts observed by the granters of the certificates are concerned, rests on the declaration of the patient that he felt an impulse to suicide. This may or may not have been the case, but there is no doubt that a Sheriff by refusing his order on such certificates would have incurred a very serious responsibility. At the same time, however, it is not impossible that the patient may have feigned a tendency to suicide, for the purpose of being declared insane, with some ulterior object in view. Three instances have occurred since the institution of the Board, in which it was alleged that insanity was feigned for the purpose of evading legal responsibility; and reasons of considerable weight were brought forward in support of this belief. It is confessedly occasionally a matter of extreme difficulty to determine whether insanity is real or feigned; and this is especially the case where the mental affection is described as a liability to morbid impulse."

Other difficulties also arise from the necessity of the Sheriff's authority being required before a lunatic can be admitted into an asylum, and in concluding the first portion of their Report, the Commissioners say:—

"We have been induced to dwell thus fully on the difficulties which complicate the working of the present statute, with the view of showing the inexpediency of requiring a too rigid adherence to fixed forms in disposing of the insane. It appears to us that a Public Board might safely be entrusted with the power to modify the schedules necessary for the purpose, provided that no effect should be given to such modification until it was approved of by the Home Secretary.

"In Scotland, the general procedure in regard to lunatics approaches much too closely to that of the criminal law, and the necessity of obtaining the Sheriff's order for placing a patient in an asylum, frequently exercises a powerful influence in deterring friends from having recourse to this measure. Many persons, too, who would voluntarily place themselves under treatment are prevented taking this course by their unwillingness to submit to the present forms."

The question of the true value of the Sheriff's interposition before a lunatic can be placed under care in an asylum, is one of great importance at the present moment to England as well as Scotland, for it has been proposed to introduce a somewhat similar method of procedure in this country. We shall not hesitate, therefore, to detail certain opinions upon the subject recently expressed by Dr. Christison.* He conceives that the objections made to the Sheriff's interference, to wit, that it is (1) an obstacle which occasions injurious delay in some urgent cases of insanity; (2) that it keeps up, through the interposition of a law officer—who happens, among other functions, to have to discharge those of a criminal judge—that unfortunate prejudice in society which is too apt to regard an asylum as a prison rather than an hospital; and (3) that it carries with it no advantage that will outweigh these drawbacks,—these objections, he conceives, will not bear scrutiny.

"Admitting," he writes, "that injurious delay does really now happen so often as to require being prevented in future, there is a plain and easy remedy in extending to a period of three days the shorter term, for which at present, by virtue of a special certificate of urgency, a lunatic may, before the Sheriff's decision, be confined on certification of his insanity by two medical practitioners. The mistake that the public is prone to commit, of confounding a lunatic asylum with a

---

* See the introduction to his pamphlet *On some of the Medico-Legal Relations of the Habit of Intemperance.* Edinburgh, 1861.

prison, has no necessary connexion that I can see with the fact that a legal official of the Crown must authorize admission, but depends simply on the more general facts that admission is compulsory on the subject of it; and the mistake will, therefore, be equally apt to recur, whether the final authority for admission be given by a sheriff's warrant, a lunacy commissioner's sanction, or a physician's bare certificate. The third objection, that the sheriff's part in the form of admission into an asylum serves no useful purpose, has been founded, I fear, on a hasty view of the question, and deserves fuller consideration.

"No one conversant with the history of asylum law and practice in this country can fail to have observed how much more satisfied and sound the tone of the public mind is in Scotland than in England on the subject of restraint on account of insanity. In England there has been for some time a timorous dread of iniquitous confinement; it is no uncommon event for actions at law, charging unjustifiable and malicious confinement, to prove successful; and the morbid feeling of the country has plainly led juries to take sometimes a prejudiced and unfair view against the defenders on such occasions. In Scotland there is no fear, on the part of the public, that sane persons can be confined as insane; but, on the contrary, great confidence that impartial justice is rendered to all. Actions for erroneous confinement are rare, founded on frivolous pretences, and sometimes in themselves no small confirmation of insanity. Accordingly, no such prosecution, so far as I am aware, has hitherto found a jury on its side in any trial in Scotland."

Hence, Dr. Christison argues, the interposition of a legal functionary of high standing between the medical certificate and the asylum, is a protection both to the profession and the public, and each has an important interest in maintaining the integrity of the procedure.

Dr. Christison's opinions upon this question carry unusual weight, and we shall content ourselves with quoting them without comment. The points at issue are chiefly matters of fact, the true significance of which must be determined by observation bearing directly upon the doubts raised by Dr. Christison. For the solution of these doubts we shall look forward with confidence to the subsequent reports of the Commissioners in Lunacy.*

* We have just received the report of a most important meeting of the medical profession of Scotland, held in Edinburgh on the 7th of June, for the purpose of memorializing the Lord-Advocate as to certain modifications and amendments of the Scotch Lunacy Act. The proceedings of this meeting were of unusual interest, particularly in reference to the treatment of excessive intemperance and the Sheriff's jurisdiction in cases of insanity, and they will be found *in extenso* at the close of the present number of this Journal. The following resolution, confirmatory of the opinions entertained by the Scotch Commissioners in Lunacy, and in opposition to those of Dr. Christison, upon the intervention of the Sheriff, was adopted by a large majority:—

"That the existing form of the Sheriff's jurisdiction in cases of insanity is objectionable, as tending to delay and obstruction in the admission of cases of urgency into asylums, and also as interfering with the physician's province, which is to judge of the circumstances under which treatment in asylums is required; and that the security of the public requires only that the competency and good faith of the

The space at our disposal forbids us following in detail the rest of the Commissioners' Report. A few jottings taken here and there must suffice.

Notwithstanding the uncertainty in which the question of further legislation still remains, several of the District Lunacy Boards are taking active steps for building asylums. The returns of expenditure for pauper lunatics in 1859 support the conclusion derived by the Commissioners from those of 1858, that "it was for the interests of the ratepayers, as well as for the welfare of the patients, to restrict, as much as possible, the use of poorhouses for the cure and treatment of insane patients." The Statistical Section of the Report is of considerable interest, and promises well for the subsequent great value of the Scotch Statistics of Lunacy. Commenting upon the accumulation of chronic cases in asylums, the Commissioners remark:—

"The detention of the insane in these establishments should be a matter of thoughtful consideration in each individual case, and the question which the superintendent should seek to determine is, not whether the insanity of a patient is such as to warrant his continued detention, but whether it is such as to render his discharge impossible or impolitic. We are strongly impressed with the conviction that it is highly desirable to bring the propriety of detaining patients in asylums periodically under review; and we are therefore of opinion that the authority for their detention should be periodically renewed. The law of Holland authorizes the detention of a patient at first only for a period not exceeding three months, and afterwards from year to year, on satisfactory evidence that adequate reasons exist, beyond mere unsoundness of mind, for warranting prolonged detention."—(p. xxxi.)

The following observation is also worthy of note:—"We have had occasion to note a large number of cases of religious melancholia and excitement during the past year, which was generally ascribed to the influence of revivalism; but to what extent this cause has been productive of insanity, or has merely modified its symptoms, we are without the means to determine."

The influence exercised by the Commissioners in ameliorating the condition of single patients is illustrated by several most interesting cases. We read of one single patient chained to his bed for upwards of thirty years; of another sleeping habitually in the same compartment with a pig; of a third kept in a cage for several years, in a small back room of a shop, in a state of absolute nudity. These cases, with others, were saved from further horrible maltreatment and neglect by the interference of the

---

medical men signing the certificate should be placed beyond suspicion; and that the Lord-Advocate be requested to take these circumstances into his consideration, with a view either to the amendment of the Sheriff's jurisdiction, or to its being replaced by some other provision for accomplishing the object in view."

Board. The condition of individual asylums is, as usual, illustrated by extracts from the reports of the Visiting Commissioners. It is painful to record that the public have failed to take any warm interest in the endeavours made last year for the purpose of establishing a national institution in Scotland for the training and education of idiots. The Commissioners estimate that there are 2236 idiots and imbeciles in Scotland, of whom about 270 are ascertained to be below 15 years of age. The condition of the insane in poor-houses is passed carefully under review, and with results highly unfavourable to the continued reception of lunatics into them.

The Report terminates with an Appendix, chiefly containing many valuable statistical tables, and the General Reports on the Condition of Single Patients made by the Visiting Commissioners during the year 1860. These Reports are of extraordinary interest, and afford an insight perhaps not otherwise attainable into the social habits and condition of several of the least known districts of Scotland. As contributions also to our knowledge of the influence exercised by insanity within the spheres of domestic and social life, it would be difficult to exaggerate their value. Of the different reports, perhaps the English reader will be most interested with Dr. Browne's on Argyll, and Dr. Mitchell's on Lewis. We regret we cannot transfer these graphic papers bodily to our pages, as well as much of the other and equally valuable reports. We must content ourselves with a quotation from Dr. Mitchell's report, illustrative of the calamitous consequences of insanity in social life.

"The force of the calamity," he writes, "in all its aspects and ramifications which insanity inflicts on society, can never be better seen than during the visitation of single patients. On this and on other points, a single parish is sometimes marvellously instructive. Take C., for instance, in which there were found six pauper lunatics living singly.

"The first I saw was a poor woman labouring under melancholia, passing into fatuity. For seventeen of the eighteen years of her insanity, she has been kept lying on a shake-down in a dark corner of a gloomy room, never seeing a brighter object than a dingy wall. There she has lain till permanent flexure of her legs has resulted, with complete inability to walk. I found her a pale emaciated sufferer, weeping and moaning for the loss of her daughter, who had been for many years her nurse, and who had very recently died of consumption, exhibiting also symptoms of melancholia before her death. There is reason to believe, that the early judicious treatment of this case would have led to recovery. As it is, nothing remained but to take from her life as much of its misery as possible; and steps were immediately adopted to brighten the atmosphere in which she lives, and to surround her with the conditions of health.

"My next visit was to a poor orphan idiot, to whom had fallen the worst of all heritages. He was one of three idiots, the children of a father who died insane, and of whose relatives several were idiots.

"The third patient was an idiot woman, who, before she was fifteen years of age, bore an illegitimate child, also an idiot, but now dead.

"I next saw a young lad of 24, a complete idiot, the bastard child of an idiot mother, not now alive. From the day of his birth he has been a pauper, and he will continue to be so till that of his death.

"My fifth visit was paid to a loathsome, slavering idiot, who, twelve years ago, bore an illegitimate child, still alive and sane.

"It has often occurred to me that distressing cases of this nature can only be made to cease by making the administrators of the criminal law bound to investigate them, and by placing in their hands the power of punishment.

"The last of the six was a helpless, speechless idiot, the child of parents who were full cousins.

"Or take another parish, K., where there are also six pauper lunatics. Three of these labour under melancholia, and of all these a 'love disappointment is the assigned cause. One has a brother and sister insane, one has a father and uncle insane, and another a nephew. One, a deformed idiot, is the child of full cousins, and she has an idiot sister, a lame sister, and a paralytic brother. Another is the child of second cousins. While another has borne three illegitimate children. (p. 252).

It would be well if we could have somewhat similar reports to these from several English districts.

---

